# An Energy-Efficient Routing Protocol with Reinforcement Learning in Software-Defined Wireless Sensor Networks

**DOI:** 10.3390/s23208435

**Published:** 2023-10-13

**Authors:** Daniel Godfrey, BeomKyu Suh, Byung Hyun Lim, Kyu-Chul Lee, Ki-Il Kim

**Affiliations:** Department of Computer Science and Engineering, Chungnam National University, Daejeon 34134, Republic of Korea; danielgodfrey2@o.cnu.ac.kr (D.G.); 202250241@o.cnu.ac.kr (B.S.); lbhzzang@o.cnu.ac.kr (B.H.L.)

**Keywords:** SDWSN-IoT, multi-objective routing, reinforcement learning, energy-efficient routing

## Abstract

The enormous increase in heterogeneous wireless devices operating in real-time applications for Internet of Things (IoT) applications presents new challenges, including heterogeneity, reliability, and scalability. To address these issues effectively, a novel architecture has been introduced, combining Software-Defined Wireless Sensor Networks (SDWSN) with the IoT, known as the SDWSN-IoT. However, wireless IoT devices deployed in such systems face limitations in the energy supply, unpredicted network changes, and the quality of service requirements. Such challenges necessitate the careful design of the underlying routing protocol, as failure to address them often results in constantly disconnected networks with poor network performance. In this paper, we present an intelligent, energy-efficient multi-objective routing protocol based on the Reinforcement Learning (RL) algorithm with Dynamic Objective Selection (DOS-RL). The primary goal of applying the proposed DOS-RL routing scheme is to optimize energy consumption in IoT networks, a paramount concern given the limited energy reserves of wireless IoT devices and the adaptability to network changes to facilitate a seamless adaption to sudden network changes, mitigating disruptions and optimizing the overall network performance. The algorithm considers correlated objectives with informative-shaped rewards to accelerate the learning process. Through the diverse simulations, we demonstrated improved energy efficiency and fast adaptation to unexpected network changes by enhancing the packet delivery ratio and reducing data delivery latency when compared to traditional routing protocols such as the Open Shortest Path First (OSPF) and the multi-objective Q-routing for Software-Defined Networks (SDN-Q).

## 1. Introduction

The dramatic and exponential increase in the urban population has created a huge demand for supplying high-quality communication services to its citizens [1]. Simultaneously, recent technological advancements have led to a significant increase in smart electronic devices, such as sensors, actuators, smartphones, and smart appliances, thereby creating new business opportunities for providing high-quality communication services. Effective communication among this vast number of electronic devices is possible through the Internet of Things (IoT), a ubiquitous network of interconnected objects that can interact with the physical world and use existing Internet standards to provide services for information transfers, analytics, and applications [2]. The design and deployment of such sophisticated systems, which entail digitalizing all aspects of our lives, face enormous challenges that require careful consideration, such as networking, security and privacy, intelligent data analysis, and smart sensors [3]. Over the years, traditional wireless sensor networks (WSNs) have evolved into becoming IoT devices capable of providing diverse sensing services in IoT systems [4]. However, wireless IoT devices inherit the characteristics of the traditional WSNs, which inhibit their application in IoT systems due to their energy-constrained behavior. To meet the application-specific requirements of the IoT in real-time, the energy cost for transmission poses a challenge in IoT applications and should be thoroughly considered [5].

IoT-based wireless sensors are deployed in computationally demanding and energy-constrained environments, necessitating the exploration of solutions that enable the prolonged operation of wireless sensor nodes without requiring battery replacements or location changes [6]. Among the various tasks performed by such nodes, data transmission emerges as the most energy-intensive, followed by others such as route computation, idle listening for potential incoming traffic, and data processing [7]. As a result, the development of effective routing protocols that identify optimal routes for a successful data transfer with acceptable energy consumption is critical for enhancing the overall performance of IoT-oriented WSNs. Despite the significant number of existing routing protocols in the literature, it is worth noting that current solutions are relatively limited in their ability to address the energy efficiency challenges of IoT-oriented WSNs. However, the integration of emerging networking technologies, such as Software-Defined Networks (SDN), into wireless sensor networks has shown promising results in the development of effective energy-efficient schemes. By removing energy-consuming tasks such as routing, data processing, and network management from the physical wireless nodes, the SDN has transformed them into data forwarding entities, significantly reducing their energy consumption [8]. The SDN architecture, with its centralized management approach that separates control logic from network devices, eliminates distributed operations and provides a holistic view of the network, better reflecting the actual network conditions. Leveraging its ability to collect comprehensive network information and create a global network view, the SDN architecture has facilitated the introduction of new optimization techniques, such as artificial intelligence and machine learning (ML) algorithms capable of solving complex problems [9].

In this study, we investigate the integration of Reinforcement Learning (RL), a  machine learning algorithm, with SDN technology to propose a novel multi-objective energy-efficient routing protocol for IoT-oriented WSNs. The resulting model, named SDWSN-IoT, comprises a multi-layered architecture with multiple controllers working collaboratively to ensure the flexible management and monitoring of network operations. Moreover, we extract network knowledge to facilitate the intelligent routing of collected data. The primary contributions of this paper can be summarized as follows:

- Identify the limitations of current RL-based routing schemes.
- Propose an intelligent multi-objective routing protocol named DOS-RL for SDWSN-IoT.
- Present distinctive features of the proposed routing scheme.
- Implement and evaluate the performance of the proposed solution in comparison to the OSPF and SDN-Q routing schemes in terms of energy efficiency and other related parameters.

The rest of this chapter is organized as follows. Section 2 provides a comprehensive review of recent studies on routing schemes for SDWSN, with a particular focus on RL-based routing schemes and architectures in the context of an intelligent SDWSN. Drawing from the insights gained in this review, we identify the limitations of existing studies and propose our intelligent multi-objective energy-efficient routing scheme for the SDWSN. Section 3 delves into the design and implementation details of our proposed scheme, offering a thorough examination of its various components. Furthermore, it provides an in-depth analysis of the unique characteristics that distinguish our routing scheme, including the introduction of shaped rewards during learning and the Dynamic Objective Selection algorithm. Section 4 showcases the experimental results and conducts a comprehensive performance evaluation of our proposed framework. Lastly, in Section 5, we conclude the paper and discuss potential future works.

## 2. Background and Related Work

This section begins with a concise background and overview of recent studies focusing on the optimization of energy consumption in the SDWSN. We conclude this section by reviewing recent research efforts on machine learning-based energy-efficient routing schemes applied in this domain.

### 2.1. SDWSN-IoT

In recent years, the Internet of Things (IoT) has emerged as one of the most influential technologies of the 21st century. The IoT encompasses a wide range of devices, from everyday household objects to sophisticated industrial tools, that can exchange data with other devices and systems via the internet [10]. The sharp increase of IoT devices, connected through both wired and wireless networks, has experienced exponential growth and is projected to surpass 22 billion by 2025 [11]. This extensive network holds the potential to optimize the reliability, resilience, and efficiency of IoT systems that are applicable in many areas, including agriculture, transportation, energy, homes, health, industry, and infrastructure [12,13,14]. However, the diverse nature of SDWSN-IoT devices still faces a number of challenges, such as security, deployment flexibility, and the absence of efficient energy consumption schemes [15]. To effectively tackle these challenges, the integration of SDWSN-IoT emerges as a pivotal concept, facilitating the establishment of a robust platform for resolving the aforementioned concerns.

**Figure 1:**
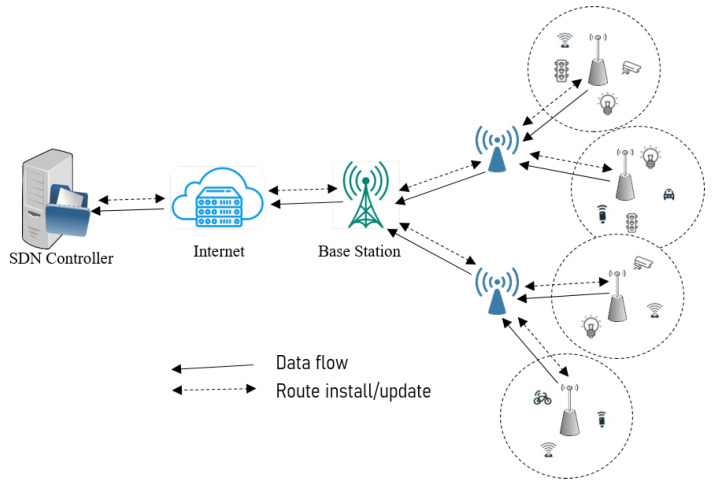
The traditional SDWSN-IoT architecture.

The SDWSN-IoT framework operates by separating the control logic from the network devices, leaving the sensing devices with only data-forwarding functionality. With  the introduction of SDWSN-IoT in networking, tasks that demand a large amount of computing power, such as routing, data processing, and network management, are removed from the physical sensor nodes and handled at the control plane by the SDN controller. As depicted in Figure 1, a typical SDWSN-IoT framework comprises multiple layers, namely the sensing, data, and control layers. Each layer consists of specific devices performing assigned tasks. The lower layer, known as the sensing layer, is composed of deployed IoT wireless sensor objects. These devices are responsible for collecting raw data from the environment and delivering it to the data layer. The  data layer comprises devices capable of forwarding the collected data to the IoT cloud. With enhanced computational capacity, memory, and an unlimited power supply, the controller in the control layer assumes the crucial role of managing data flow across the forwarding devices in the data layer. The seamless flow of the substantial amount of collected data relies on the data-forwarding rules, which are installed on the forwarding sensor nodes by the SDN controller [16]. By using protocols such as Sensor OpenFlow [17], the controller efficiently manages the dynamic flow of collected data in real-time by regularly computing optimal routing paths based on the real-time network status. By decoupling the control layer from the data layer, the  SDWSN-IoT framework has enabled the programmable control and virtualization of the network functionalities.

The SDWSN-IoT paradigm is undeniably a promising and pivotal technology in wireless IoT applications. However, effectively collecting and routing a substantial amount of data through energy-constrained IoT sensor devices remains a significant challenge. Therefore, the development of an energy-efficient routing scheme is crucial for enhancing the overall performance of such networks. The design of such a routing scheme determines the longevity of the network, directly influencing the duration of active data flows across multiple paths. The integration of IoT applications and the SDWSN framework has facilitated the adoption of advanced and resource-intensive intelligent optimization techniques that were previously unattainable [18]. These techniques include resource-demanding intelligent machine-learning algorithms that require considerable energy, computational power, and memory supply to operate effectively [19].

In recent years, the incorporation of machine learning algorithms into the SDWSN has drawn substantial attention from the community, as it introduces the concept of task automation in SDWSN-IoT [20]. Task automation in the SDWSN includes a wide range of solutions such as classification, the prediction of future network conditions, and the installation of optimization rules based on measured network conditions. The integration between machine learning algorithms and SDWSN-IoT architecture is a promising approach to optimizing the energy consumption of sensor nodes, mainly for the following reasons:

- Adaptive Optimization: Machine learning algorithms can analyze real-time network conditions and historical data to make intelligent decisions for optimizing energy consumption in IoT applications. By dynamically adapting to changing network conditions, the algorithms can optimize the operation of sensor nodes, reducing unnecessary energy consumption [21].
- Intelligent Routing: Machine learning algorithms can learn from past routing patterns and make predictions about future network conditions. This enables them to intelligently route data, considering factors such as energy constraints, network congestion, and node availability. By choosing efficient routing paths, energy consumption can be minimized while maintaining effective data transmission [22,23,24].
- Proactive Resource Allocation: Machine learning algorithms can analyze data patterns and predict resource demands in advance. This allows for proactive resource allocation, ensuring that resources, such as energy, computational power, and memory, are efficiently allocated to meet the demands of IoT applications. By  optimizing resource allocation, energy efficiency can be significantly improved [25].
- Anomaly Detection: Machine learning algorithms can detect anomalies and abnormal behavior in the network. By identifying unusual patterns or events that may lead to energy wastage or inefficiency, proactive measures can be taken to mitigate such issues and optimize energy efficiency in IoT applications [26].
- Predictive Maintenance: Machine learning algorithms can analyze sensor data and predict potential failures or maintenance requirements. By proactively addressing maintenance needs, energy can be saved by preventing unexpected downtime and optimizing the overall performance of IoT devices [27].

The following subsection introduces the commonly applied ML algorithm applied to find optimal routes for the intelligent forwarding of collected data across the network in IoT applications.

### 2.2. Reinforcement Learning-Based SDWSN-IoT

Unlike traditional routing algorithms, machine learning algorithms provide a distinct advantage as they are capable of surpassing the static nature of forwarding rules in flow tables and adapting to the dynamic network environment, enabling the identification of crucial flow items with remarkable accuracy. Machine learning algorithms offer a remarkable advantage by enhancing network performance through automated tasks and the ability to predict future network conditions. Addressing the need for minimized network latency and energy efficiency within the constraints of application dependence in a software-defined fog-IoT network, a  study [28] introduced a dynamic task scheduling and assignment approach based on deep reinforcement learning (DRL). This method formulates the task assignment and scheduling problem as an energy-constrained Deep Q-Learning process, yielding promising results. In the realm of large-scale IoT networks, a  study [29] investigated and proposed an SDN model with a Q-routing algorithm to efficiently route large data while reducing energy consumption. The proposed scheme showcased significant improvements in terms of the delivery latency, packet delivery ratio, and energy consumption. To optimize the overall processing delay within an energy-limited environment of a cloud-edge terminal collaboration network, which encompasses mobile-edge computing and an SDN architecture, a study [30] introduced a reinforcement learning-based joint communicational and computational resource allocation mechanism (RJCC). This approach demonstrated an enhanced performance in terms of the average energy consumption and average data delivery latency for communicating IoT devices.

Furthermore, several studies have explored the application of machine learning algorithms in finding optimal data transfer routes within SDN-based network architectures for the SDWSN. For instance, a study [31] introduced an intelligent architecture for self-learning control strategies in software-defined networks by proposing a routing scheme based on deep reinforcement learning (DRL). This scheme, known as NetworkAI, utilizes deep reinforcement learning techniques and leverages network monitoring technologies like inband network telemetry to dynamically generate control policies, leading to near-optimal decision-making. Similarly, in [32], the authors presented a scheme to optimize the routing path in the SDWSN using the Reinforcement Learning (RL) algorithm. Their approach incorporates energy efficiency and network Quality-of-Service (QoS) parameters into the reward function. The proposed routing scheme compares various SDN-based techniques, including the traditional SDN, energy-aware SDN (EASDN), QR-SDN, TIDE, as well as non-SDN-based techniques such as Q-learning and RL-based routing (RLBR). The results indicate that the RL-based SDWSN outperforms other approaches in terms of the network lifetime and packet delivery ratio.

This paper introduces a routing scheme that aims to enhance the capabilities of SDWSN-IoT by integrating cognitive machine learning techniques, leveraging the inherent features of SDWSN-IoT such as network programmability and comprehensive topology monitoring. The proposed framework facilitates dynamic learning and the adaptation to network changes, enabling the proactive installation and continuous updating of routes based on rapidly changing link states, thereby ensuring that swift and efficient routes are found for data forwarding.

## 3. Methodology

The integration of intelligent machine learning-based algorithms harnesses the advantages offered by SDWSN-IoT architecture and is considered a promising solution for optimizing the network performance. By intelligently acquiring and utilizing real-time network data, these algorithms can determine optimal routes for the efficient transfer of network traffic while conserving network resources [33]. This section provides a comprehensive introduction to the unique characteristics and emerging technical issues that have guided the design of our proposed energy-efficient routing scheme for the SDWSN-IoT network architecture.

We begin by identifying a critical concern that is often overlooked when designing multi-objective energy-efficient RL-based routing protocols and present a proposed solution. The key issues identified include the slow learning process, conflicts among multiple objectives, and the uncertainty of learned values, which can result in additional overhead during the learning process. Further details on the proposed routing scheme are provided below.

### 3.1. Preliminary

Reinforcement learning algorithms involve a learning agent interacting with its environment to find and select actions based on the current state and feedback signals received to optimize a predefined objective. These algorithms rely on reward functions, which provide feedback signals to guide the learning process by evaluating the effects of taken actions. However, designing an appropriate reward function is not trivial, as it can be challenging to guide the learning agent without getting trapped in local optima [34]. Consequently, even seemingly solvable reinforcement learning scenarios may produce unexpected results. The goal of reinforcement learning is either to maximize the expected return of a reward signal or to minimize costs for optimization problems, indicating how well the learning agent performs [35]. The  RL reward signals can be classified into two categories: sparse and dense rewards. Sparse rewards provide feedback only at the end of a learning episode, indicating whether the agent succeeded or failed in its task. On the other hand, dense rewards give feedback at every stage of the decision-making process, making them more effective for learning compared to their sparse counterparts. However, learning based on sparse rewards can be time-consuming as the agent needs to explore more to collect rewards and learn the optimal policy. Traditional multi-objective RL optimization approaches frequently combine dense rewards linearly and reduce them to a single scalar objective using methods like the weighted sum [36]. While this approach aims to guide the learning agent in progressing toward its goal, it often necessitates intensive weight tuning to set a priori preferences among objectives.

In this study, we utilize ’reward shaping,’ incorporating heuristic knowledge from the system designer to provide learning agents with additional reward signals. These extra rewards enable the agent to differentiate between good and bad actions early in the learning process, encouraging exploration in areas of the state space that hold promising solutions. Such prior knowledge can be integrated into the reward function or initial agent values. Despite often deploying reinforcement learning agents without prior information, previous research [37] has demonstrated the benefits of providing heuristic knowledge in guiding exploration. Our study shows that designing meaningful reward shapings leads to faster and improved solutions.

### 3.2. Correlated Multi-Objective Energy-Efficient Routing Scheme for SDWSN-IoT

While most decision problems actually have multiple objectives, most algorithms dealing with agents that need to interact with sequential decision problems focus on optimizing a single objective. Dealing with multi-objective problems (MOP) requires the simultaneous optimization of multiple feedback signals or objectives. Most research on multi-objective optimization is focused on solving problems with conflicting objectives, and rightly so, as these are hard problems with many possible Pareto optimal trade-off solutions. To nonetheless deal with the multiple objectives of the real world, a common approach to creating decision-theoretic agents is to combine all the important aspects together into a single, scalar, additive reward function. The  scalarization functions involve assigning an individual weight to *m* objectives, allowing the reward function designer to have control over the trade-off of the objectives. This trade-off is parametrized by $w_{o}\in \left[0,\;1\right]$ for objective *o*, in  a linear combination $\sum_{o=1}^{m}w_{o}\cdot Q_{0}\left(s,a\right)$ whereby $\sum_{o=1}^{m}w_{o}=1$.

This iterative process, which is done repeatedly until the behavior is acceptable, lacks explainability of the decision-making process and is often subjected to a retraining process when preferences between objectives change. Furthermore, assigning weights to objectives is a challenging task that often demands extensive weight tuning and a thorough comprehension of each objective’s preference. Failure to properly set these weights often results in biased or suboptimal solutions, highlighting the complexity involved in achieving an effective objective weighting strategy. For example, when trying to train an agent in a network environment, the  reward function designer may wish to double the weight of one objective. Even when the objectives are linearly weighted in the reward function, doubling the weight of one objective for a specific aspect of performance might not guarantee a better performance of that objective due to the potential non-linear relationship between the reward weights and the actual objective outcomes.

Applying typical *RL* frameworks to determine optimal routing paths for multi-objective optimization, while using a vector of conflicting objectives as a reward function, presents challenges in managing trade-offs among the objectives, which can lead to suboptimal routing paths. For instance, in the context of routing paths for multi-objective optimization, conflicting objectives may include minimizing delay and maximizing throughput. Improving delay might involve choosing longer paths with less congestion, while maximizing throughput may require selecting shorter paths that could be more congested. Achieving a balance between such competing objectives is challenging since enhancing one objective often comes at the expense of sacrificing performance in another. The study focuses on identifying multi-objective solutions with correlated objectives, where the limited Pareto front makes it challenging to discern clear trade-offs. The reward function designer prioritizes the fast discovery of a near-optimal policy and its closeness to optimality over distinguishing very similar optimal trade-offs. To optimize the performance, a reinforcement learning agent leverages both signals in combination rather than relying solely on one. To  address this, the study transforms a single-objective MDP into a *Correlated Multi-Objective Markov Decision Process* (CMOMDP) by incorporating multiple potential-based shaped reward functions.

In this study, we have chosen to implement the CMOMDP approach due to its ability to address multiple correlated optimization objectives, providing comprehensive information about the system. The primary objective of achieving energy efficiency is the optimization of the average energy consumption of nodes. We define energy efficiency of the network as the ability of the network to perform its intended functions and tasks (e.g., data transmission and processing) while minimizing the use of energy resources. This objective has been expanded to include several other correlated objectives that necessitate simultaneous consideration, such as load balancing and reliability. Optimizing load balancing is crucial because balancing data traffic loads across the network can help prevent some nodes from depleting their energy quickly, prolonging the network’s overall operation. Reliability, on the other hand, pertains to the ability of the communication link to consistently deliver data packets without errors or failures. To optimize the reliability objective, we focus on selecting links with good quality since a higher link quality usually leads to increased reliability. When the link quality is strong and stable, the chances of data packets being transmitted accurately and successfully are higher. Given these insights, optimizing both load balancing and the link quality is pivotal in enhancing the energy efficiency and overall functionality of SDWSNs-IoT.

Therefore, optimizing any of the objectives mentioned above is expected to have a positive impact on the network’s energy efficiency. As such, our proposed scheme revolves around the concept of dynamic objective selection, which takes into consideration that certain objectives may hold greater relevance or reliability in specific states compared to others, tailoring the decision-making process accordingly. Further details on the proposed dynamic objective selection scheme are provided in the next subsection below.

#### 3.2.1. Dynamic Objective Selection RL with Shaped Rewards

Dynamic Objective Selection (DOS)-RL is an approach that combines the principles of Q-learning, a model-free reinforcement learning technique, with the concept of dynamic objective selection. In DOS-RL, the traditional Q-learning algorithm is extended to dynamically select objectives during the learning process based on the current state of the system. The DOS-RL learning agent dynamically selects an objective to optimize from a predefined set of objectives based on its confidence in the estimated Q-values. This means that only the estimated Q-values of the most confident objective are considered when taking an action, allowing the agent to adaptively choose the objective it believes is the most promising at that given time. The Q-learning technique operates by iteratively estimating the action-value (Q-value). Q-learning is easy to implement and applicable to solving many problems since the estimation of the Q-value is independent of the policy that the learning agents follow. In Q-learning, the agent learns sequentially through a series of stages known as epochs. For example, in epoch *n*, the agent is in state $s_{n}$, performs action $a_{n}$, and receives an immediate reward $r_{n}$ as it transitions to the next state $s_{n+1}$. The action-value is updated at discrete time *t* according to the equation below:

(1) $$Q\left(s_{t},a_{t}\right)\leftarrow \left(1-\alpha \right)\cdot Q\left(s_{t},a_{t}\right)+\alpha \cdot \left[R_{t+1}+\gamma \cdot \max_{\forall a\in A}\left\{Q\left(s_{t+1},a\right)\right\}\right],$$

whereby α is the learning rate to determine how fast the agent learns. The discount factor γ determines how much the learning agents take into consideration rewards in the distant future, relative to those in the immediate future, and *R* is the reward received for performing an action *a* at time *t* while in the state $s_{t}$ resulting in the new state $s_{t+1}$.

As previously mentioned, the Dynamic Objective Selection (DOS) scheme operates by the parallel estimation of the action-value for each objective *o*, based on the measured current state *s* and a potential future state $s^{\prime }$. The action selection decision in DOS Q-learning is based on the objective that the learning agent determines to have the highest level of certainty in its estimated Q-values. This dynamic approach highlights the adaptive nature of objective selection, as the agent prioritizes objectives based on its confidence in the estimated Q-values. The  foundation of this idea can be traced back to the work by Brys et al. [38], where they introduced the Adaptive Objective Selection (AOS) method for handling correlated objectives in multi-objective RL for pathfinding problems. To quantify the confidence in Q-values, they assumed a normal distribution and utilize the mean of the estimated Q-values to track the variance of the distribution using the TD-error $\delta_{o}$ for objective *o* as follows:

(2) $$VAR\left(s,a,o\right)\leftarrow \left(1-\psi \right)\cdot VAR\left(s,a,o\right)+\psi \cdot \delta_{o}^{2}.$$

Algorithm 1 presents the pseudocode for the DOS approach. When the agent intends to select an action, it first determines which objective it has the most confidence in, based on a confidence metric $C_{k}$. It then makes an action selection decision, using the ϵ-greedy strategy, based solely on the estimates for that particular objective. The variance reflects the dispersion of the action-value distribution for each objective. The confident estimation of an objective will produce uniform values, which leads to a small variance σ. In contrast, a larger variance indicates that the policy is more unstable for objective *k*. We define the confidence metric $C_{k}$ in Equation (3) to mathematically formulate these rules as follows:

(3) $$C_{k}=\frac{K(\mu_{k}+e^{\sigma_{k}^{2}})}{\sum (\mu_{k}+e^{\sigma_{k}^{2}})}.$$

Notably, the DOS is scale-invariant due to the nature of the statistical tests proposed, meaning it does not depend on any differences in scaling between the objectives. Furthermore, the DOS operates automatically without any parameter introductions, making it a fully self-contained mechanism.
**Algorithm 1** Dynamic Objective Selection (DOS)**for** each objective *o* **do**    $C_{k}\leftarrow \mathrm{confidence}\left(\left(s,a_{1},o\right),\ldots ,\left(s,a_{n},o\right)\right)$**end for**$o_{\mathrm{best}}\leftarrow \underset{k}{\arg\min}\;C_{k}$actionSelection($Q\left(s,a_{1},o_{\mathrm{best}}\right),\ldots ,Q\left(s,a_{n},o_{\mathrm{best}}\right)$)

#### 3.2.2. Components and Architecture of the Proposed DOS-RL for the SDWSN-IoT Scheme

**Figure 2:**
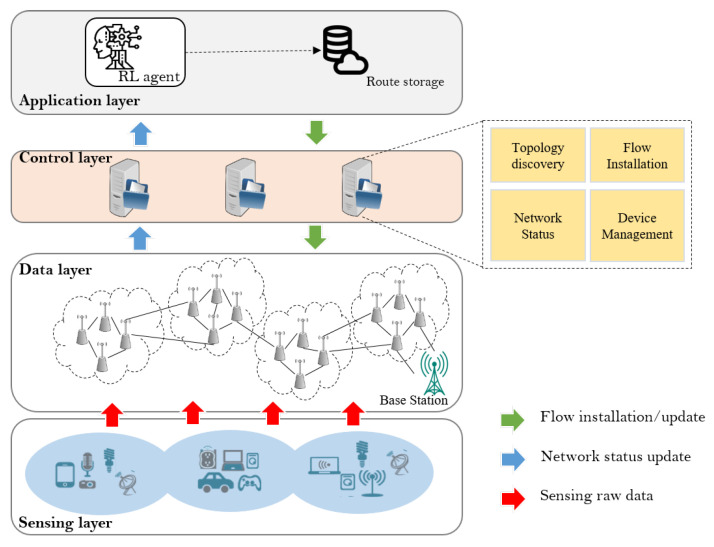
Architecture of the proposed DOS-RL routing scheme for SDWSN-IoT.

Prior to introducing the architecture of the proposed multi-objective energy efficiency routing scheme, it is essential to understand the components contributing to its functionality. The following subsection provides a comprehensive description of the proposed scheme, as depicted in Figure 2. Given the substantial deployment of a vast number of sensor nodes in IoT networks, an effective solution is needed to address scalability and management challenges. To tackle this, we implement an SDN architecture as the underlying framework for the multi-objective energy efficiency routing scheme. This hierarchical structure ensures optimized decision-making and coordinated communication, thereby facilitating the achievement of energy-efficient routing in a resource-constrained IoT network environment.

The proposed architecture consists of four layers: the sensing layer, the data layer, the control layer, and the application layer. The sensing layer comprises various IoT sensing devices that are extensively deployed to gather sensing data. Meanwhile, the data layer is composed of wireless sensor nodes capable of performing a multi-hop transmission. These nodes facilitate the transfer of a substantial volume of data collected by the sensing nodes (SNs) in the sensing layer and relay it to the base station (BS). The proposed architecture incorporates the IEEE 802.15.4 [39] protocol without any modifications to the existing hardware platform, ensuring a seamless integration with the network sensor devices. The control layer of the system is composed of four key modules: the *Topology discovery*, *Network status*, *Flow installation*, and  *Device management* module.The four modules are implemented by the network controller (NC) to constantly monitor the status of relay nodes (RNs) on the data layer. By implementing the SDN architecture, resource-intensive tasks such as routing, data aggregation, and network management are removed from the RNs leaving them with only data forwarding functionalities. By default, the RNs have the capability to sense and collect data from the SNs, and subsequently, forward it to the BS. We suggest that the RNs with residual energy less than a certain threshold should not be part of routing. Furthermore, we adopt a strategy of deploying multiple distributed controllers instead of relying on a single controller framework to enhance control overhead and accelerate the network configuration speed.

The application layer of the proposed framework incorporates an intelligent agent that leverages intelligent techniques to transform raw network information into actionable knowledge. The primary objective of our proposed routing scheme is to automate traffic routing based on network information, aiming to achieve multi-objective energy-efficient routing. To accomplish this, our scheme employs the RL to determine the optimal routes for all source-destination pairs, taking into account several correlated link-state metrics related to the energy efficiency of the RNs such as residual energy, estimated link quality, and queue congestion. By leveraging a centralized network view, our routing scheme dynamically adapts to traffic changes and adjusts its routing strategies accordingly. Shaped rewards are employed in the RL to guide the selection of appropriate behaviors for adaptive routing, enabling the effective handling of changes in network conditions. The computed routes are stored in the route repository, accessible to the flow installation module of network controllers (NCs). This proactive approach allows the module to populate the flow tables of the RNs in advance for all anticipated traffic matches. A sample route entry would be represented as follows: (source = n1, destination = n30, next hop = 14, route lifetime = 3 s). The inclusion of route lifetime information ensures that a node does not use the same route indefinitely but periodically uses newly updated route information. By default, NCs are programmed to send topology update messages (TUs) to the RNs at regular intervals. However, in certain scenarios, such as unexpected packet loss, TUs may be sent as urgent route update messages from the RNs.

#### 3.2.3. The DOS-RL Routing Scheme

The realistic conditions of an SDWSN-IoT network are subjected to dynamic changes in resources such as battery capacity, CPU capacity, memory, and bandwidth, as well as link quality changes during network operation. RL-based routing schemes have demonstrated considerable advantages in designing network operation policies that can handle such changes. However, such routing protocols often fail to respond quickly enough to such changes. To address this issue, we propose the DOS-RL routing scheme which allows the RL agent to learn from multiple correlated objectives simultaneously and to adaptively choose the objective it believes is the most promising from the current state. Therefore, we have turned the problem of energy efficiency into an RL process by modeling it into a four-tuple(S, A, P, R), defining the states (S), actions (A), policy (P), and reward functions (R) of the DOS-RL scheme.

1. *The Reward Function (R)*: In reinforcement learning, the agent evaluates the effectiveness of its actions and improves its policy by relying on rewards collected from the environment. The rewards obtained are typically dependent on the actions taken, with varying actions resulting in differing rewards. To  implement the proposed DOS-RL scheme, we define three reward functions, each corresponding to one of the correlated objectives: the selection of routing paths with sufficient energy for data forwarding ($o_{1}$), load balancing ($o_{2}$), and  the selection of paths with a good link quality for a reliable data transfer ($o_{3}$). Route selection based on any of these objectives is expected to improve the energy efficiency of the IoT network system. Details on each objective of the DOS-RL scheme and its related reward functions are provided below:
  (a) Energy-consumption: To achieve objective $o_{1}$, we consider the energy-consumption parameter, which is a critical factor in determining the overall energy efficiency of an SDWSN: for instance, in a scenario where node *i* forwards a packet to node *j*. The reward received by node *i* for selecting node *j* as its relay node in terms of energy consumption is estimated by the reward function $R\_E_{i,j}$ for the state-action pair, $\left(s_{i},s_{j}\right)$ as:
    (4) $$R\_E_{i,j}=Remaining\_Energy\left(s_{j}\right)-Remaining\_Energy\left(s_{i}\right)$$
    where $Remaining\_Energy(s_{j})$ represents the remaining energy of node *i* in state $s_{i}$ as a percentage; meanwhile, $Remaining\_Energy(s_{i})$ represents the remaining energy of node *j* in state $s_{j}$ as a percentage. The formula calculates the difference in remaining energy between node *i* and *j* after the data transmission. A higher value of $R\_E_{i,j}$ indicates that node *i* has consumed less energy in forwarding the packet to node *j*, which is desirable to achieve the energy efficiency objective $\left(o_{1}\right)$.
  (b) Load balance: To optimize energy efficiency and network performance in the SDWSN, the careful selection of relay nodes and balanced workload distribution are crucial. Otherwise, some nodes along overused paths may become overloaded, leading to bottlenecks and delays, which can result in a degraded network performance. For objective $o_{2}$, we utilize the parameter-available buffer length to estimate the degree of queue congestion in relay nodes. The reward $R\_Q_{i,j}$ for load balancing as observed by node *i* when selecting node *j* is computed as follows:
    (5) $$R\_Q_{i,j}=\frac{Available\_Buffer\_Length(s_{j})}{Max\_Buffer\_Length}\;-\frac{Load(s_{i})}{Max\_Load}\;$$
    where $Available\_Buffer\_Length(s_{j})$ represents the available buffer length of node *j* in state $s_{j}$, Max_Buffer_Length represents the maximum buffer length of a node, $Load(s_{i})$ represents the current load of node *i* in state $s_{i}$, and Max_Load represents the maximum load capacity of a node. This formula considers both the available buffer length of node *j* and the current load of node *i*. The second term subtracts the load of node *i* from the load balancing reward value, allowing for a more comprehensive estimation that takes into account both node *i* and node *j* in the load balancing process. A higher value of $R\_Q_{i,j}$ indicates a better load balancing situation for the given state-action pair $\left(s_{i},s_{j}\right)$.
  (c) Link quality: A wireless link can be measured by retrieving useful information from either the sender or receiver side. To achieve $o_{3}$, we use simple measurements to estimate the link quality based on the parameter packet reception ratio (PRR), measured as the ratio of the total number of packets successfully received to the total number of packets transmitted through a specific wireless link between two nodes. Unlike other sophisticated techniques, this approach involves a low computation and communication overhead. Instead of using an instant value of the PRR, we calculate an average-over-time using an Exponentially-Weighted Moving Average (EWMA) filter. Suppose node *i* is forwarding data packets to node *j*, the reward $R\_LQ_{i,j}$ received by node *j* based on PRR using EWMA is estimated as follows:
    (6) $$R\_LQ_{i,j}=PRR\_est_{i,j}\left(k-1\right)\cdot \alpha_{EWMA}+PRR\_sample_{i,j}\left(k\right)\cdot \left(1-\alpha_{EWMA}\right),$$
    whereby $PRR\_est_{i,j}\left(k-1\right)$ is the previously estimated average, $PRR\_sample_{i,j}\left(k\right)$ is the most recent measured value of the packet reception ratio calculated, and $\alpha_{EWMA}$ is the filter parameter.
2. *The State Space (S)*: We define the state space as a graph corresponding to the global topology created by the RNs on the data plane, as seen by the intelligent RL controller. Each state in the state space corresponds to an RN, and a state transition refers to a link connecting two RNs. The intelligent RL controller uses the partial maps created by the *topology discovery module* to create a global topology. Therefore, the cardinality of the set of states depends on the number of nodes that can actively participate in routing.
3. *The Action Space (A)*: The action space, denoted as A, includes all possible actions that an agent can undertake from a given state of the RL environment. It defines the choices available to the agent at each time step, presenting the range of options to the agent. In our specific problem, the  discrete action space comprises a finite number of actions that the RL agent can select when in a particular state $s_{i}\in S$. The cardinality of *A* at state *i* is determined by the number of nodes eligible to participate in the routing process from that specific state.
4. *The Optimal Policy (P)*: The policy determines how the learning agent should behave when it is at a given state with the purpose of maximizing the reward value in the learning process. Our proposed scheme estimates the Q-function of every objective *o* simultaneously and decides, before every action selection decision, which objective estimate $o_{best}$ an agent will consider in its decision-making process. We use the concept of *confidence*  on computed Q-values by representing each action as a distribution and using a normal distribution of Q-values and the mean values to keep track of the variance as shown in Equation (2). The agent approximates the optimal Q-function by visiting all pairs of action-states and stores the updated Q-values in the Q-table. In our proposed scheme, the  approximated Q-value $Q(s_{t},a_{t})$ represents the expected cumulative reward when the RL agent is in the state $s_{t}$ and takes action $a_{t}$, transitioning to a new state $s_{t+1}$ while maximizing the cumulative rewards for an objective $o_{n}$, where n∈N and *N* denote the set of all objectives. The Q-learning equation to update Q-values is designed as shown below in Equation (7):
  (7) $$Q^{n}\left(s_{t},a_{t}\right)\leftarrow \left(1-\alpha \right)\cdot Q^{n}\left(s_{t},a_{t}\right)+\alpha \cdot \left[R_{t+1}^{n}+\gamma \cdot \max_{\forall a\in A}\left\{Q^{n}\left(s_{t+1},a\right)\right\}\right].$$
  To steer the exploration behavior of the learning agent by incorporating some heuristic knowledge on the problem domain, we introduce an extra reward $F_{t+1}$ onto the reward received from the environment $R_{t+1}$. The newly added shaping reward function *F* is included when updating the Q-learning rule as follows:
  (8) $$Q^{n}\left(s_{t},a_{t}\right)\leftarrow \left(1-\alpha \right)\cdot Q^{n}\left(s_{t},a_{t}\right)+\alpha \cdot \left[R_{t+1}^{n}+F_{t+1}+\gamma \cdot \max_{\forall a\in A}Q^{n}\left(s_{t+1},a\right)\right].$$
  To avoid changes on the optimal policy, F is implemented as the difference of some potential value, $F_{t+1}$ = γϕ(t+1)−ϕ(t) over the state space, where ϕ is a potential function that provides some hints on states. In our study, we define ϕ as the Normalized Euclidean Distance between the current state(s) and the goal state(G) expressed as:
  (9) $$\phi =1-\frac{\mathrm{distance}(S_{t},G)}{\max_{\forall x\in S}\left(\mathrm{distance}\left(x,G\right)\right)},$$
  where $distance(S_{t},G)$ is the Euclidean distance between the current state $S_{t}$ and the goal state *G*, and $\max_{\forall x\in S}\left(\mathrm{distance}\left(x,G\right)\right)$ represents the maximum distance between any state *x* in the state space *S* and the goal state *G*. The potential function guides the agent towards the goal state by giving bigger rewards to states that are closer to the goal and smaller rewards for states that are farther away.
5. *In DOS Q-routing*: To find the best action-value of the Q-function, the learning agents use an action selection mechanism to trade-off between the exploitation and exploration of available action space. To achieve this, our proposed scheme uses the ϵ-greedy exploration and exploitation method, whereby ϵ∈[0, 1], allowing the agent to exploit with probability *pr* = ϵ and explore with probability *pr* = 1 −ϵ. The agent action selection is determined by a randomly generated number x∈[0, 1], of which if x<ϵ, the agent exploits it by taking an action that returns the most expected optimal value; otherwise, it explores it by selecting a random action based on the most confident objective $o_{best}$ as observed from the current state by the learning agent as shown below:
  (10) $$\mathrm{actionSelection}=\left\{\begin{array}{cc} \max_{a\in A}\left\{Q\left(s,a,o_{\mathrm{best}}\right)\right\}, & \mathrm{if}\;x<\epsilon \\ \mathrm{randomAction}(Q\left(s,a,o_{\mathrm{best}}\right),\cdots ,Q\left(s,a_{n},o_{\mathrm{best}}\right)), & \mathrm{otherwise} \end{array}\right..$$

#### 3.2.4. DOS-RL Routing Algorithm

The proposed energy-efficient routing scheme employs the DOS-RL scheme with shaped rewards to acquire knowledge about the network environment. Algorithm 2 outlines the procedure employed in the proposed approach. The algorithm aims to find optimized routing paths for all source-destination pairs between the current state and the goal state for all valid link states.
**Algorithm 2** The DOS-RL with Shaped Rewards Routing Scheme1:**Input:**2:Learning rate: α3:Exploration and exploitation parameter: ε4:Number of learning episodes: *n*5:Potential function for each state: ϕ6:All link pairs (src,dst): VLinksPair7:Link-states, Set of learning objectives: N8:Initialize *Q*: Q(S,A)=0, ∀s∈S, ∀a∈A9:**procedure** $S_{j}\left(G\right)\leftarrow \mathrm{STP}(\mathrm{VLinksPair})$▷ Set of loop-free paths from the initial state to the end state *G*10:    **for** each objective *o* in N, compute Q-values for all (src,dst) in $S_{j}\left(G\right)$ **do**11:        **for** episode←1 to *n* **do**12:           Start in state $s_{t}=\mathrm{src}\in S$;13:           **while** $s_{t+1}$ is not dst **do**14:               Select $A_{t}$ for $s_{t}$ using the ε-greedy method according to Equation (10)15:               $R_{t}^{{}^{\prime }}\leftarrow R_{t}+F_{t+1}$ ▷ Get the shaped reward $R_{t}^{{}^{\prime }}$ and observe new state $s_{t+1}$16:               Update $Q^{n}\left(s_{t},a_{t}\right)$                             ▷ Update according to Equation (8)17:               $s_{t}\leftarrow s_{t+1}$                            ▷ Set the current state $s_{t}$ as the new state $s_{t+1}$18:           **end while**19:        **end for**20:        Take *Q*-table and find the path between src and dst with the maximum Q-values21:    **end for**22:    Store the set of computed paths in the route repository23:**end procedure**

Stages followed by this algorithm can be summarized as follows:

1. *Initialization and Setup*: The intelligent controller is initialized and assigned an IP address. Partial topologies received from the controllers are used to create a global topology containing the eligible RNs. Set the learning rate α, discount factor γ, and exploration rate ε, and define the potential function ϕ to provide hints on states for shaping rewards (Lines 2 to 7).
2. *Loop-Free Route Calculation*: Loop-free routes are computed using the Spanning Tree Protocol (STP) algorithm for all given source-sink pairs. This step ensures that the Q-learning algorithm focuses only on exploring states that lead to the end goal. Here, a source node is defined as any RN that receives data from the SN(s) at the sensing layer (Line 8).
3. *Q-Routing Exploration*: The set of computed loop-free routes serves as input for the SDN intelligent controller, which uses the Q-routing algorithm to find the best path for routing. The Q-learning process runs for a specified number of episodes or until the final state is reached. For each state $S_{t}$, compute Q-values for every action $a_{t}$ for each objective *o* simultaneously, based on the current Q-table values (Lines 9 to 13).
4. *Q-Learning Process*: During the learning process, the RL agent dynamically chooses the learning objective from the current state, takes the next action, and moves to the next state. Regarding the randomly generated number *x* ranging between 0 and 1, according to the exploration and exploitation ε-greedy method, if *x* < ε, then exploit with the probability pr=ε by selecting the action with the highest Q-value for the most confident objective $o_{best}$. Otherwise, explore with the probability pr=1−ε by randomly selecting an action for the most confident objective $o_{best}$ (Lines 14 to 19).
5. *Optimal Route Determination*: The RL agent uses the generated Q-table to determine the most rewarding route between the source and sink nodes. This is based on the state-action combinations that received the highest Q-values after completing a transition. Finally, all found optimum routes are stored and sent to respective controllers. These routes are installed or updated in the routing table of the RNs for immediate use in the network.

#### 3.2.5. Algorithm Time Complexity Analysis

When dealing with RL-based routing protocols, making the time-complexity of that algorithm is crucial for reasons such as: making the performance analysis and determining if the algorithm is suitable for the intended application; resource allocation; and assessing how well the algorithm scales with the increasing problem size, real-time, and dynamic environment requirements. Study [40] suggests that the time complexities of RL algorithms require updating the Q-table of each state $\forall s_{t}^{i}\in S_{i}$ for each state in the state space *S*. This means, in a worst-case scenario, the time complexity of the RL-based algorithm is when the learning agent visits all the states in the state and action spaces. This suggests that the time complexity of RL algorithms depends on the size of the state space. The state space size is $O(N^{2})$, where *N* could be the number of nodes in the network, the amount of traffic between a source node and a destination node (traffic matrices), or static vectors. The size of the action space is limited to the *k* potential paths associated with each state. The complexity for RL is, then, $O(kN^{2})$, where *k* is a constant.

## 4. Performance Evaluation

This section presents an evaluation of the proposed DOS-RL routing scheme. The chapter is organized into multiple sections and subsections, which outline the simulation tools and frameworks used to measure the scheme’s performance. Furthermore, it include details about the test environment, performance metrics, learning parameter settings, and the observed results, which will be presented and discussed.

### 4.1. Simulation Environment

To evaluate the effectiveness of our proposed scheme, we conducted simulations using ns-3 [41], an open-source system-level network simulation tool capable of creating an environment for the easy transfer of states and actions between AI frameworks and the ns-3 simulation environment. To achieve this, we employ the ns3-ai [42] module, which enables seamless integration between ns-3 and open-source AI frameworks like TensorFlow and PyTorch by utilizing shared memory. The ns3-ai module consists of two components: the ns-3 interface, implemented in C++, and the AI interface developed in Python. These components work together to ensure the fast and efficient exchange of large data volumes from a C++ program to a Python program. By utilizing shared memory, the ns3-ai module facilitates communication between multiple processes. Unlike the ns3-gym framework [43], which relies on pipes or sockets, the use of shared memory allows for the creation of a highly efficient core module for data transfer.

**Figure 3:**
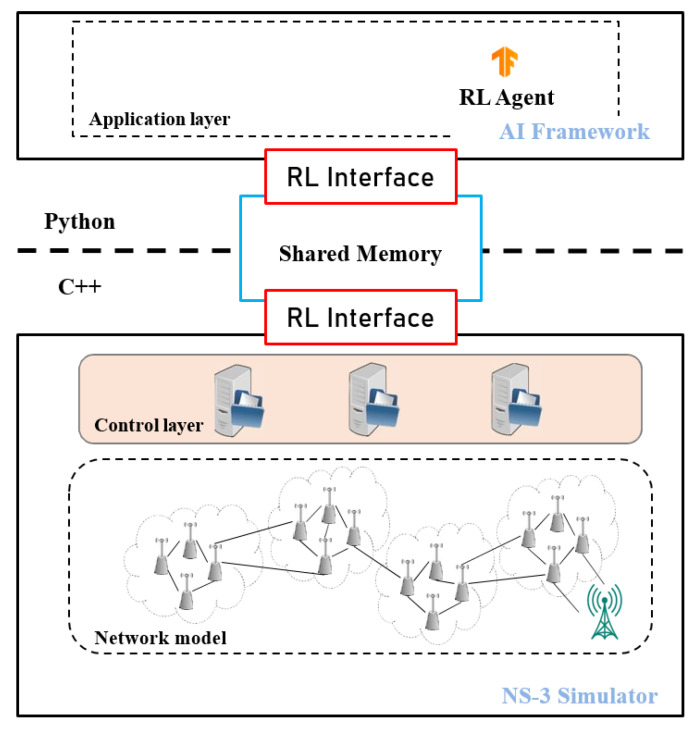
DOS-RL SDWSN Architecture.

To implement our proposed scheme, we have designed a system architecture that combines the ns3-ai and the SDWSN architecture, as shown in Figure 2. The proposed architecture, depicted in Figure 3, consists of two main components: the ns-3 simulator and the AI framework. The ns-3 simulator serves as the data generator by providing environments to create simulation scenarios. It generates relevant information, which is then fed into the AI framework for training the model to make real-time decisions. The AI framework processes the data received from the ns-3 simulator and trains the model to make intelligent decisions. The shared memory pool facilitates the seamless data exchange between the ns-3 and the AI framework, allowing both sides to access and manipulate the data. Control signals, managed by four modules operating at the control layer, ensure smooth communication and coordination between the ns-3 and the AI framework. This integration enables efficient decision-making and the evaluation of our proposed routing scheme.

### 4.2. Simulation Settings

In subsequent sections, we detail our experimental setup and the default simulation parameters. Our primary focus is to evaluate the performance of the DOS-RL routing algorithm using three widely-accepted network performance metrics. Additionally, we compare its performance against two state-of-the-art routing protocols: OSPF and SDN-Q. For our evaluations, we establish a network with a varying number of wireless sensor nodes randomly distributed within a fixed-size simulation area. Each sensor node has the capability to choose multiple relay nodes for data forwarding. We installed traffic source applications on a varied number of nodes, ranging from 2, 4, 6, 8, to 10. This diverse setup allows for a comprehensive analysis of the DOS-RL routing algorithm’s performance across various network scenarios.

To determine the optimal paths from each source node to the target node, we employ the Q-learning algorithm to evaluate the set of all available loop-free paths. These paths are identified by the STP algorithm for a specified pair of source-destination nodes. Our experimentation begins by comparing the performance of different RL-based routing schemes. This includes the DOS-RL without shaped rewards, DOS-RL with shaped rewards (DOS-RL Shaped), and the traditional RL which utilizes a linear combination of multi-objectives based on the weighted sum approach (RL). The objective of our experiments is to observe and compare their performance on a pathfinding problem in a five by five dynamic Gridworld environment.

Key performance indicators for evaluating these algorithms include their convergence speed by observing the average rewards collected; average energy consumed, which reveals how efficient the agent is in conserving energy while completing the task; the average episode length which shows the average number of steps taken by the agents to complete episodes; and finally, the average frequency of hitting obstacles which indicates how well the agents avoid obstacles in the environment. During experiments, the reward function of the RL includes three objectives: reducing the number of steps taken to reach the goal state, avoiding obstacles, and optimizing charging points (optimizing energy consumption). The DOS-RL with shaped rewards incorporates the additional shaping reward function based on the Manhattan distance to the goal state in order to guide the learning process and speed up the learning process. The experiment design integrates varied states such as states with obstacles, states with relatively higher energy consumption, recharging states, and normal states. The learning agent starts with a five-energy unit reservoir and loses energy by 0.1 units at each regular state transition. Encountering any of the dynamically selected obstacles (of a maximum of three after every 100 episodes) results in a penalty of 20 units. Conversely, navigating through a recharge point yields a reward of five units. An agent that runs out of energy while navigating incurs a penalty of 20 units.

The agent’s navigation starts at the state (0,0) with the ultimate objective of reaching the goal state (4,4) through the shortest and most energy-efficient path. This controlled setting allows us to examine the performance of these algorithms in a well-defined scenario. The values of all important parameter settings used during the experiment are summarized in Table 1.

**Figure 4:**
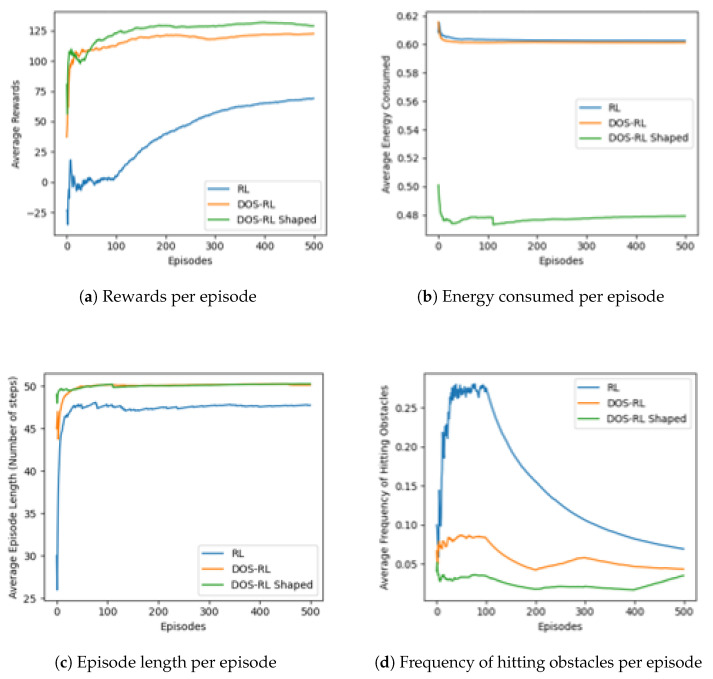
Performance comparison of different RL versions on a Dynamic GridWorld environment.

To evaluate the convergence speed of the algorithms, Figure 4a plots the learning curve for the average rewards collected and the average energy consumed per number of episodes in Figure 4b. In Figure 4c,d, we can see plots for the average episode length and the average frequency of hitting obstacles per number of episodes during the entire simulation time. In Figure 4a, we can see how the poor performance of the RL algorithm is affected by its frequency of hitting obstacles during the first 100 episodes. The DOS-RL with shaped rewards outperforms the rest of the algorithm by proving its efficiency in conserving energy (Figure 4b) by nearly 20% with the highest average collected at a cost of a slightly longer episode length compared to the RL algorithm. In summary, unlike the traditional RL, the DOS algorithms seem to perform much better by allowing the agent to explore different strategies that cater to different objectives. This allows the algorithms to find a balance between objectives more effectively.

### 4.3. Simulation Parameters Setup

The simulation settings (see Table 2) for the experiments performed were made to evaluate and compare the performance of our proposed routing scheme. We ran the Q-learning algorithm (see Algorithm 2) to compute optimal paths for given source-destination pairs under varied network conditions, such as the number of traffic sources and the number of RNs in the network of the size of a 1500 by 1500 m area. To get an accurate estimate of the simulation results, we run each simulation scenario 10 times to obtain the averaged results. To simplify the experiments, we also make the following assumption for the RNs in the network: all the nodes have the same flow table size, same buffer size, start with the same initial energy, and have the same transmission/reception power and communication range.

### 4.4. Performance Evaluation

In order to assess the performance of the routing methods, we have selected three network metrics: the (1) packet delivery ratio (PDR), (2) end-to-end delay (E2E), and (3) energy efficiency (EE). These metrics hold significant importance when evaluating the network, particularly in terms of the impact caused by path selection on the energy consumption of the wireless nodes. We define these metrics as follows:

1. *PDR*: This refers to the ratio of successfully delivered packets to the total number of packets sent within the network. It is a measure of the effectiveness of the routing protocols and communication infrastructure in ensuring that packets reach their intended destinations without loss or errors. A higher PDR indicates a more reliable and efficient network, whereas a lower PDR suggests potential issues such as packet loss, congestion, or faulty routing. Monitoring and optimizing the PDR is crucial in evaluating and improving the overall performance and reliability of the WSNs.
2. *E2E*: This is the time it takes for a packet to travel from the source node to the destination node. It includes processing, queuing, transmission, and propagation delays. A lower delay is better for faster and more efficient communication. Minimizing the end-to-end delay is crucial in the WSNs to ensure timely and reliable data delivery.
3. *EE*: This refers to the ability of the network to perform its intended tasks and communication while utilizing minimal energy resources. We defined this metric as the ratio of the PDR to the average energy consumed by the RNs. It is a critical consideration in the WSNs due to the limited and often non-rechargeable energy sources available to the sensor nodes. By enhancing energy efficiency, the WSNs can achieve a longer network lifetime, extended monitoring capabilities, and reduced maintenance requirements.

In the following subsections, we will compare and discuss the performance of the proposed scheme alongside its counterparts: the traditional OSPF and SDN-Q based on the network metrics.

#### 4.4.1. Packet Delivery Ratio of Routing Protocols

**Figure 5:**
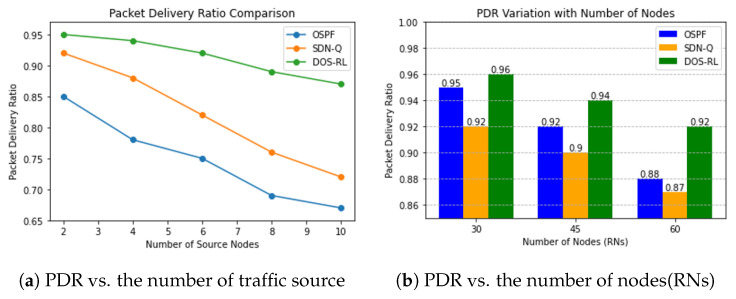
Packet delivery ratio performance.

In our evaluation, we examine the performance of three routing protocol schemes in a network environment comprising 30 randomly distributed Relay Nodes (RNs). We investigate how the Packet Delivery Ratio (PDR) of these schemes is affected when varying the number of traffic sources and the relay node density. Figure 5a illustrates the results, which clearly indicate that our proposed scheme outperforms the OSPF routing protocol by a significant margin. Specifically, our scheme achieves a PDR improvement of approximately 10% to 20% for conditions with both low and high traffic rates. Unlike the OSPF, which prioritizes shorter paths without considering real-time network conditions, the DOS-RL and SDN-Q are capable of applying intelligent decision making which achieves a relatively better performance. The better performance exhibited by the proposed DOS-RL compared to that of the SDN-Q is due to its adaptability features to the dynamic changing network environment assisted by shaping rewards, which assists it in making decisions somewhat carefully to avoid poor energy consumption by dynamically adjusting objectives based on the measured networking conditions to avoid congestion and minimize packet loss.

Next, we explore the impact of varying the number of deployed nodes (RNs) in the network while keeping other parameters, such as the number of traffic sources, constant at five. Interestingly, we observe that the difference in the PDR remains relatively small across the different numbers of the RNs. In Figure 5b, we notice a gradual decline in the PDR as the number of nodes increases. This decline can be attributed to factors such as increased network complexity, scalability, congestion, and increased overhead resulting from a larger number of nodes sharing the network resources. Nevertheless, our proposed scheme continues to outperform the other routing schemes by maintaining a comparatively higher PDR, even when the number of RNs is doubled.

#### 4.4.2. End-To-End Delay of Routing Protocols

**Figure 6:**
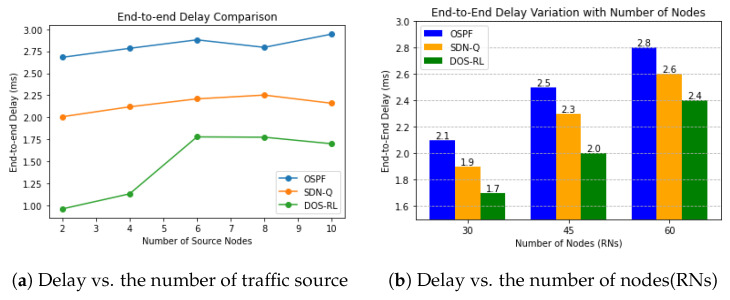
End-to-end delay performance.

Next, we look at the E2E simulation results of the three protocols as shown in Figure 6a. The OSPF routing algorithm typically aims for a low routing delay by selecting the shortest path which is expected to perform well under normal conditions; however, the results state otherwise. From the results, it can be seen that the OSPF has the worst performance. This is due to the fact that most of the initial routes selected by the OSPF algorithm do not change and are subject to being overused and fail to respond to real-time network changes. As a result, the RNs along such paths receive a larger amount of traffic than they can handle and process in a minimum time. The DOS-RL algorithm outperforms the SDN-Q not only due to its dynamic adaptability to changes, but because it can intelligently route traffic while considering different objectives such as prioritizing latency-sensitive states, optimizing energy efficiency or maximizing reliability, and making decisions to meet the specific needs of the network at a given time. Unlike the DOS-RL, traditional Q-routing is subjected to struggle to adapt to the dynamic changes in the network environment or to optimize objectives beyond what is initially defined. While the number of the RNs was varied, as shown in Figure 6b, the performance of all schemes in terms of delivery delay falls progressively. As more nodes are added to the network, the search space and hence path length increases and this affects the performance of the networks, especially during the early exploration stages for the SDN-Q and DOS-RL algorithms. However, our proposed scheme adjusts well by adjusting learning objectives in real-time. The SDN-Q fails to perform well because the paths it selects last longer and hence, increase the probability for congested paths to occur.

#### 4.4.3. Energy Efficiency of Routing Protocols

**Figure 7:**
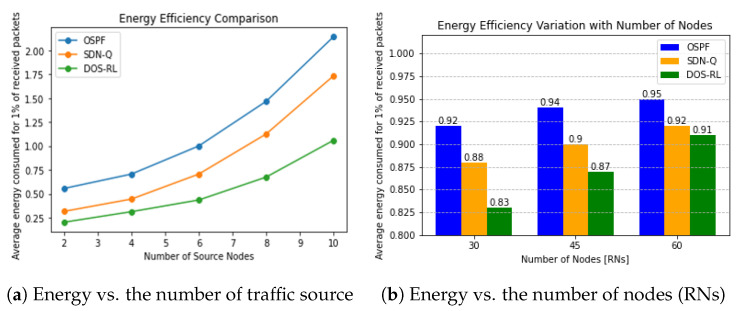
Energy efficiency performance.

Measured as the ratio of the percentage of packets successfully delivered to the average of total energy consumed during the entire simulation time, improving this parameter is one of our highest concerns. As it can be seen in Figure 7a, the performance of all three routing schemes gradually drops due to the decrease in the packet delivery ratio and the corresponding increase in average energy consumption during transmission as traffic flow increases. Regardless, the proposed scheme, with its ability to consider energy-efficient objectives, has the potential to outperform the OSPF and Traditional RL in terms of energy consumption. By selecting objectives that prioritize energy efficiency, the DOS-RL can route traffic through paths that minimize energy usage. As the simulation results exhibit, the DOS-RL scheme achieves a successful transmission of data packets to the sink node with a lower average energy consumption. By leveraging the DOS algorithm and incorporated shaped rewards, the scheme provided is capable of adjusting the objective priority while meanwhile selecting relatively shorter paths, unlike those selected by the OSPF and SDN-Q scheme. The DOS-RL scheme effectively monitors node energy levels, detects energy depletion or bottlenecks, and redirects traffic along more energy-efficient paths. This adaptability allows it to align its objectives with the energy efficiency goals of the network, surpassing the other two routing schemes.

Furthermore, as shown in Figure 7b, the DOS-RL algorithm exhibits better energy efficiency in successfully delivering data packets compared to the counterparts. With an increasing number of nodes, more nodes participate in routing which affects the average energy consumption. Additionally, factors such as the increased overhead and interference contribute to a decrease in the packet delivery ratio, impacting the overall energy efficiency of the network. Among the considered algorithms, OSPF performs the worst, as it operates without considering real-time network conditions and solely selects the shortest path, which is not always the most efficient choice.

## 5. Conclusions

In summary, our proposed routing protocol, DOS-RL with shaped rewards, offers a dynamic objective selection mechanism that optimizes the energy efficiency of SDWSN-IoT networks. By allowing for adaptive routing objectives based on correlated objectives, our approach significantly enhances the network operation efficiency. The inclusion of additional shaping rewards speeds up the learning process, surpassing the performance of traditional Q-Learning and OSPF algorithms in SDWSN-IoT routing. Leveraging the power of DOS-RL in intelligent routing, our scheme discovers reliable and energy-efficient routing policies, yielding a remarkable performance. One notable benefit of our approach is its ability to incorporate various performance metrics related to Quality of Service (QoS) requirements into the reward function. This inclusion not only accelerates learning but also enables the system to make well-informed routing decisions, reflecting specific QoS needs.

The implications of our research are profound. Our proposed routing protocol holds the potential to revolutionize SDWSN-IoT networks by significantly enhancing energy efficiency and the overall network performance. It opens doors to applications that demand efficient routing, reduced energy consumption, and reliable communication. As for future work, we are committed to further investigating the impact of different parameters on the performance of the DOS-RL algorithm. Additionally, we intend to explore alternative methods for speeding up the learning process while striking an acceptable balance among multiple objectives. These efforts aim to continually enhance the efficiency and overall performance of our proposed routing protocol in SDWSN-IoT networks.

## Figures and Tables

**Table 1:** The summary of key parameters.

Parameters	Value	Description
α	0.1	Learning rate
δ	0.99	The discount factor
ϵ	0.3	Exploration rate
φ	0.01	Minimum exploration rate
β	0.995	Exploration decay rate

**Table 2:** Simulation Parameters.

Parameters	Value/Description
Traffic type	UDP
Learning rate α	0.3
Number of RNs	30, 45, and 60
Discount factor δ	0.85
Packet size (Bits)	512
Simulation Time (s)	200
Number of source nodes	2, 4, 6, 8, and 10
Deployment of sensor nodes	Random
Packet generation rate (pkts/sec)	10
The initial energy of sensor nodes	5 Joules

## Data Availability

Available on request.
